# Applications of the second virial coefficient: protein crystallization and solubility

**DOI:** 10.1107/S2053230X1400867X

**Published:** 2014-04-30

**Authors:** William W. Wilson, Lawrence J. DeLucas

**Affiliations:** a Mississippi State University, Starkville, MS 39759, USA; b Center for Structural Biology, University of Alabama at Birmingham, 1720 Second Avenue South, Birmingham, AL 35294, USA

**Keywords:** second virial coefficient, crystallization, solubility

## Abstract

This article begins by highlighting some of the ground-based studies emanating from NASA’s Microgravity Protein Crystal Growth (PCG) program. This is followed by a more detailed discussion of the history of and the progress made in one of the NASA-funded PCG investigations involving the use of measured second virial coefficients (*B* values) as a diagnostic indicator of solution conditions conducive to protein crystallization. A second application of measured *B* values involves the determination of solution conditions that improve or maximize the solubility of aqueous and membrane proteins. These two important applications have led to several technological improvements that simplify the experimental expertise required, enable the measurement of membrane proteins and improve the diagnostic capability and measurement throughput.

## Abstract 2

This article highlights some of the ground-based studies emanating from NASA’s Microgravity Protein Crystal Growth (PCG) program, and includes a more detailed discussion of the history and the progress made in one of the NASA-funded PCG investigations involving the use of measured second virial coefficients (*B* values) as a diagnostic indicator of solution conditions conducive to protein crystallization.

## 1. Introduction   

Since the first X-ray crystallographic structure of a protein, sperm whale myoglobin, was reported in 1958 (Kendrew *et al.*, 1958 ▶), the structural biology community has generally considered the initial step, crystallization, to be more art than science. The traditional crystallization screening approach involves the preparation of a broad range of chemical conditions, often requiring several milligrams of purified protein (McPherson, 1985 ▶). Parameters screened generally include protein concentration, pH (generally a pH range from 3.0 to 9.0 is evaluated in steps of 0.3 pH units), buffer type, precipitating agent type and concentration, temperature, additive type and concentration. This approach, combined with technological developments such as high-throughput protein-expression/purification and automated crystallographic determination protocols, the use of protein engineering to enhance crystallization lattice contacts and robotic liquid-dispensing/crystallization systems, have accelerated the crystallization and structure determination of thousands of new proteins (Blundell & Mizuguchi, 2000 ▶; Burley *et al.*, 1999 ▶; Mittl & Grütter, 2001 ▶; Montelione & Anderson, 1999 ▶; Teichmann *et al.*, 1999 ▶; Christendat *et al.*, 2000 ▶; Waldo *et al.*, 1999 ▶; Terwilliger, 2000 ▶; Abola *et al.*, 2000 ▶; Hendrickson, 2000 ▶; Derewenda, 2004*a* ▶,*b* ▶; Rupp *et al.*, 2002 ▶; Krupka *et al.*, 2002 ▶; Stevens, 2000 ▶). However, in spite of the plethora of new protein structures that have resulted from these technological advances, it is clear that robotics alone is not sufficient for the successful crystallization of a significant number of important proteins. In fact, there remain thousands of important eukaryotic, prokaryotic and viral proteins that either cannot be crystallized or result in crystals of insufficient quality to enable a high-resolution X-ray crystallographic solution (Bernstein *et al.*, 1978 ▶). Low crystallization success rates are a consequence of a challenge to find the correct combinations of a large number of relevant parameters, some of which include protein polydispersity, protein purity/homogeneity, precipitating agent, buffer/pH, temperature, protein solubility, concentration, flexibility of the protein itself, protein stability and even the propensity of amino acids on the protein surface to form good protein–protein contacts. For membrane proteins and membrane-associated proteins additional factors influencing crystallization include detergent and lipid type and concentration, specific additives, protein instability and the ability to express a sufficient supply of protein.

The most recognized aspect of the NASA-sponsored protein crystal growth program involves growing crystals of macromolecules in a microgravity environment. Government-sponsored microgravity protein crystal growth programs, *i.e.* those of the US space agency (NASA), the Japanese space agency (NASDA) and the European space agency (ESA), have resulted in more than 100 peer-reviewed publications by scientists from several universities in the United States, Canada, Germany, Belgium, Spain, Italy and China. However, in addition to microgravity studies, each space agency also provided funding to support ground-based studies addressing the fundamental aspects of protein crystal growth. Some of these studies are directed at understanding the causes of different crystal defects, crystal growth termination, how crystal growth rate affects crystal quality, dynamic control of the crystal nucleation and growth phase, and investigations of how fluid flows and protein transport rate influence crystal size and quality (Li *et al.*, 1999*a* ▶,*b* ▶; Forsythe *et al.*, 2002 ▶; Gorti *et al.*, 2005 ▶; Vekilov, 2003 ▶, 2009 ▶, 2010 ▶; Booth *et al.*, 2004 ▶; Thomas *et al.*, 1996 ▶, 1998 ▶; McPherson *et al.*, 1995 ▶, 2001 ▶; Malkin & McPherson, 1994 ▶; Kuznetsov *et al.*, 1997 ▶, 2000 ▶; Drenth & Haas, 1998 ▶; Vekilov *et al.*, 1996 ▶). Other advances in protein crystallization strategy include the statistical design of experiments, analysis of screen results, automated crystal image analysis and novel seeding approaches (Luft, Snell *et al.*, 2011 ▶; Luft, Wolfley *et al.*, 2011 ▶; Nagel *et al.*, 2008 ▶; Snell *et al.*, 2008 ▶; D’Arcy *et al.*, 2003 ▶, 2004 ▶, 2007 ▶).

## 2. Review   

One area receiving little or no attention is the discovery of a method or technology that could rapidly survey solution conditions for those more likely to produce crystals for a particular protein. What is needed is a fundamental understanding of the chemical and molecular conditions necessary to produce soluble, non-aggregated, stable proteins as well as conditions that induce crystallization *versus* precipitation or nonspecific aggregation. Discovery of a crystallization assay(s) to address these issues for each new protein investigated could dramatically reduce the hundreds to thousands of different chemical conditions typically screened for each new protein. Before the advent of automated crystallization robots this task was extremely laborious, often requiring months of tedious work in the laboratory. Although crystallization robots have relieved investigators of this time-consuming task, the fact still remains that the search for crystallization conditions for a new protein typically requires that thousands of different solution conditions are screened, a process that consumes valuable protein. Trained crystallographers can be overwhelmed by the large volume of information produced by thousands of crystal screening experiments, and thereby miss subtle interrelationships between crystallization results and the numerous variables associated with crystallization experiments. Unfortunately, when crystal screens produce little to no positive results (only precipitate or clear drops) there are few leads towards potential crystallization conditions. Also, for negative results (*i.e.* clear drops) there is no information available about why a particular crystallization condition failed and how the formulation parameters influence the protein–protein interactions. In addition, once initial crystallization conditions have been discovered, finding the optimum conditions that yield high-quality crystals (and therefore high X-ray diffraction resolution) has proven to be challenging for many important proteins. Both of these issues are particularly evident for integral membrane proteins and large protein–protein complexes. A diagnostic can potentially reduce the number of experiments performed in search of initial crystallization conditions. Furthermore, once initial crystallization conditions are discovered, a diagnostic that guides optimization of the different solution components could provide a much-needed method to improve and optimize the quality of crystals.

**Figure 1:**
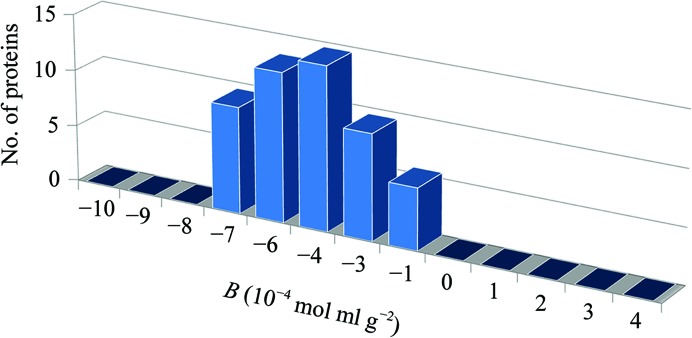
Measured *B* values *versus* crystallization conditions for 50 different aqueous proteins.

In an attempt to address these issues, Dr W. William Wilson (NASA-funded investigator at Mississippi State University) performed investigations utilizing static laser light scattering (SLS) to determine whether the second virial coefficient (*B*) could be used as a diagnostic regarding solution conditions conducive to protein crystallization. The rationale behind this hypothesis was based on the observation that slower crystal growth rates generally result in larger, higher quality crystals. The second virial coefficient, referred to as *B*, is a thermodynamic term that provides semi-quantitative information regarding the magnitude of weak protein–protein interactions in a given solvent. *B* is a measure of all of the interactions between two bodies (protein–protein), including contributions from excluded volume, electrostatic factors (attractive and repulsive) and hydrophobic interactions. In terms of McMillan–Meyer solution theory, *B* is related to a potential of mean force, *W*, which describes all of the interaction forces between two protein molecules in a dilute protein solution. Historically, the measurement of *B* for macromolecular solutions has been performed by osmotic pressure (Ruppert *et al.*, 2001 ▶; Tombs & Peacocke, 1974 ▶), sedimentation-equilibrium (Fujita, 1975 ▶) or static light-scattering (Kratochvil, 1987 ▶) experiments. One or more of these techniques are often used by pharmaceutical companies to measure the solubility of protein therapeutics (*i.e.* vaccines) in different formulations since there is a direct correlation between protein–protein attraction and protein solubility. For the purposes of a crystallization screening technology, osmotic pressure and sedimentation equilibrium are not practical, owing to both the experimental time and the sample quantity required. In 1994, the Wilson laboratory was the first to use measured *B* values, obtained using static light scattering (SLS), to demonstrate the role of protein–protein interactions in crystallization (George & Wilson, 1994 ▶). Originally determined for lysozyme and bovine serum albumin, *B* showed a narrow range of values that correlated with solution conditions that yielded crystals for each of these proteins. In similar experiments performed for each of more than 50 different proteins dissolved in a large variety of literature-reported crystallizing solvents, the compiled *B* values are shown in Fig. 1 ▶ (Wilson, 2003 ▶). The solution conditions that produced crystals for all 50 of the proteins yielded *B* values that ranged between −0.5 × 10^−4^ and −8.0 × 10^−4^ mol ml g^−2^. The Wilson laboratory also analyzed more than 300 conditions that produced values falling outside of this crystallization region (termed the crystallization slot). Those conditions yielding large negative *B* values produced amorphous precipitate, while positive *B* values resulted in clear solutions. For each protein analyzed the measured *B* values falling within the ‘crystallization slot’ (Fig. 1 ▶) were the result of solution conditions that provide gentle attraction between protein molecules. Protein crystallization experiments conducted in solution conditions at more negative *B* values have a greater risk of forming an amorphous solid phase because of corresponding stronger protein–protein attractions. Similarly, experiments yielding more positive *B* values (where the net protein–protein interactions are repulsive) typically require protein concentrations that are very high before phase separation of any kind occurs. The correlation between *B* values and crystallization conditions has been confirmed both experimentally and theoretically by a number of investigators (Rosenbaum *et al.*, 1996 ▶; Neal *et al.*, 1998 ▶; Tessier & Lenhoff, 2003 ▶; Bonneté & Vivarès, 2002 ▶; Demoruelle *et al.*, 2002 ▶; Ruppert *et al.*, 2001 ▶). An empirical correlation between *B* (a dilute solution parameter) and the solubility *s* (a phase-transition parameter) has also been demonstrated for several proteins (Demoruelle *et al.*, 2002 ▶; Gripon *et al.*, 1997 ▶; Guo *et al.*, 1999 ▶).

**Figure 2:**
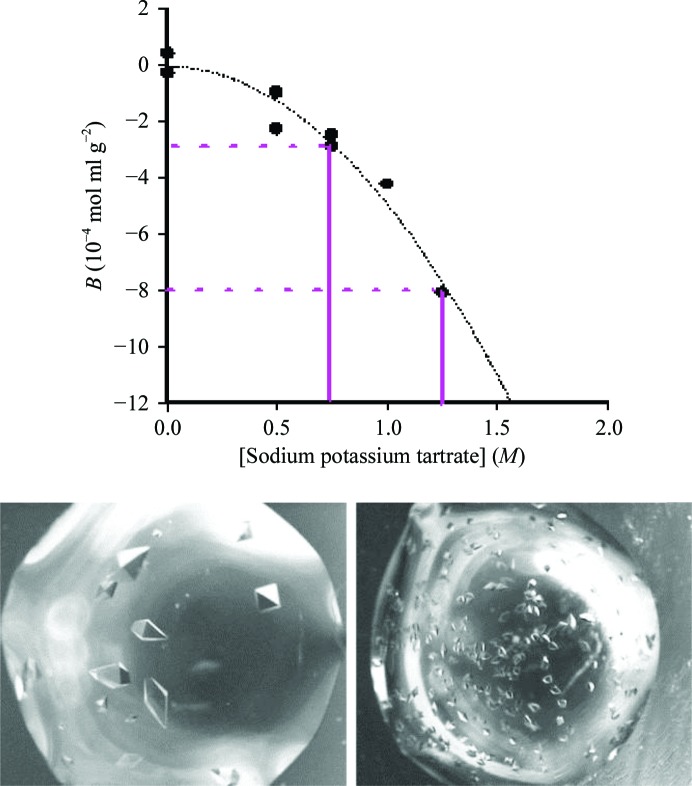
Thaumatin crystals grown at disparate *B* values within the crystallization slot.

The use of measured *B* values *via* static light scattering provides a useful diagnostic whereby solution conditions can be surveyed to determine a subset of variables and corresponding ranges of concentrations that provide gentle protein–protein attractions (George & Wilson, 1994 ▶). However, it should be noted that for proteins that have never previously been crystallized, solution *B* values that fall within the crystallization slot suggest the possibility of successful crystallization, but these conditions do not guarantee crystallization. This is owing to the fact that the protein–protein attraction, although gentle, must still involve contacts (*i.e.* hydrogen bonds and ionic interactions) that are correctly positioned to form an ordered crystalline lattice. For each protein surveyed, conditions lying outside the crystallization slot (highly negative or positive *B* values) are extremely unlikely to result in crystals. In addition to narrowing the number of crystallization experiments performed to produce crystals for a new protein, second virial coefficient values can also be used to improve or optimize initial crystallization conditions. Fig. 2 ▶ shows crystals of thaumatin grown by the batch method at different *B*-value conditions (all residing within the crystallization slot). As the *B* values become more negative, protein–protein attractive forces increase, producing more rapid crystal nucleation and growth. The result is that more but smaller crystals (and possibly more poorly ordered crystals) are produced at successively larger negative *B* values within the slot. Generally, lower negative *B* values (indicating more gentle protein attractive forces) produce fewer but larger crystals that typically grow more slowly. It has also been observed that *B* values for crystallization conditions of larger proteins (*i.e.* molecular weight greater than 60 kDa) generally fall in the −0.5 to −3.0 range of the crystallization slot, implying weaker protein attraction.

*B*-value experiments performed on approximately 70 different aqueous and membrane proteins clearly demonstrate the potential of the values as a diagnostic for initial crystallization conditions as well as for improving existing crystallization conditions (Berger *et al.*, 2005 ▶, 2006 ▶; Dumetz *et al.*, 2007 ▶; García, Hadley *et al.*, 2003 ▶; Patro & Przybycien, 1996 ▶; Pjura *et al.*, 2000 ▶; Tessier & Lenhoff, 2003 ▶; Tessier, Lenhoff *et al.*, 2002 ▶; Tessier, Vandrey *et al.*, 2002 ▶; Valente *et al.*, 2005 ▶). However, in spite of the demonstrated capability of SLS *B*-value measurements to serve as a diagnostic for solution conditions that are more likely to result in crystals, this experimental approach has not gained traction within the crystallographic community. This is owing to a number of factors, including the following: (i) the need for nano-crystallization robotic systems capable of rapidly screening several thousand crystallization conditions with less than 1 mg purified protein, (ii) SLS measurements require user expertise, (iii) SLS measurements are tedious and time-consuming and (iv) SLS measurements are extremely challenging for membrane proteins that also contain detergent/lipid molecules.

These constraints led the Wilson, Henry and DeLucas laboratories to explore self-interaction chromatography (SIC), which had previously been demonstrated as an alternative experimental approach to measure *B* values (Patro & Przybycien, 1996 ▶). The technique is similar to affinity chromatography, with differences in the interpretation of protein solution times and the preparation of column media. The following basic steps are utilized.

(i) Covalently bind the protein of interest to the chromatography medium (stationary phase) followed by loading the protein-bound medium into a column. There are four alternative chemistries available to bind the protein to the support medium (available from Tosoh Bioscience). All are relatively benign and have been successfully used for sensitive aqueous and membrane proteins.
(ii) Flow the formulation of interest over the column using standard high-performance liquid-chromatography hardware.
(iii) Inject the soluble protein (mobile phase) into the formulation flowing through the column followed by measurement of the volume required for elution of the soluble protein (retention volume) as it interacts with the same protein that is randomly bound covalently to the column medium.
(iv) Compare the retention volume measured in step (iii) with the retention volume of the soluble protein injected into a column of inert medium (often referred to as a ‘dead column’).

The SIC technique is based on the assumption that increased attraction between the injected mobile-phase protein and the covalently but randomly bound protein will result in an increase in the solution volume required to elute the injected protein from the column. The comparison of retention volumes in the presence and absence of randomly bound column protein provides a normalized retention factor. This retention factor is subsequently used to compare the magnitude of protein self-interaction in the presence of different formulation conditions. However, utilization of the SIC retention volume as a measure of protein–protein interaction in different formulations produced two problems.

(i) The thermodynamic model of the SIC experiment was not apparent, thereby preventing SIC measurements from being independently verified *via* previously established thermodynamic measures of protein–protein interactions.
(ii) Control experiments comparing the retention volume for the protein eluted from a protein-bound column with the same protein diluted from inert medium (no protein bound to the column) did not account for the excluded volume. This is problematic because protein oligomers (which often exist in purified protein preparations) would naturally have access to less volume in the column and would therefore elute faster. A reduced elution volume of strongly interacting molecules in the mobile phase is counter to the SIC premise that mobile-phase molecules should interact with the stationary phase, resulting in increased solution volume.

**Figure 3:**
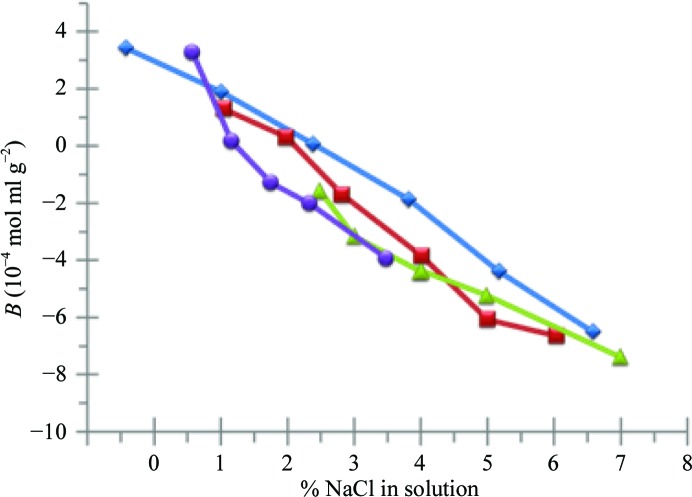
Comparison of SIC (blue diamonds) and SLS data for lysozyme as a function of NaCl concentration. The data are taken from the following references: blue diamonds, Valente *et al.* (2005 ▶); red squares, García, Holman *et al.* (2003 ▶); green triangles, Guo *et al.* (1999 ▶); purple circles, Rosenbaum & Zukoski (1996 ▶).

These issues were addressed by Tessier *et al.* (2002 ▶) *via* an appropriate thermodynamic model which incorporated a non-interacting molecule (acetone) to correct for excluded volumes, combined with protein injections over both the inert and protein-bound column media (Tessier, Lenhoff *et al.*, 2002 ▶; Tessier, Vandrey *et al.*, 2002 ▶). The retention-volume ratio between protein and acetone accounts for excluded volume contributions by the protein. Since acetone is a non-interacting marker, measurement of the acetone retention volume is periodically required in steps (iii) and (iv) to ensure that the live and dead columns have not changed physical configuration owing to medium compression or degradation of covalently bound protein. Protein molecules in solution interact *via* a variety of forces including electrostatics, dipole–dipole and van der Waals forces. McMillan–Meyer solution theory expands ideal solution theories to account for molecular interactions. The molecular interaction parameter (*B* value) can be calculated from SIC measurements using the following equations (Tessier, Vandrey *et al.*, 2002 ▶): 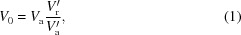


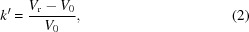


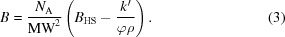



(1) is the void volume, *V*_0_, adjusted for the excluded volume of the protein. *V*_a_ is the eluted volume of the acetone injection over the protein-bound column and *V*_r_/*V*_a_ is the ratio of the protein elution volume to the acetone solution volume or the inert column. This ratio accounts for the contribution of the protein to the excluded volume. With this correction, the retention factor, *k*′, is calculated using the measured retention volume, *V*_r_, of the protein over the protein-bound column. The *k*′ value is entered directly into the equation for the *B* value (3). *N*_A_, MW, *B*_HS_, ϕ and ρ are all based on the protein and medium independent of protein–protein interactions. *N*_A_ and MW are Avogadro’s number and the molecular weight of the protein, respectively. *B*_HS_ is the excluded volume contribution by the protein as a hard sphere (calculated based on the molecular weight of the protein). Finally, the phase ratio, ϕ, is the ratio of available surface area to available volume and is an established value dependent on the medium (DePhillips & Lenhoff, 2000 ▶). A Pierce BCA (protein concentration) assay is used to determine ρ, the number of covalently bound protein molecules per unit area of column medium. The retention factor then becomes the formulation-dependent variable, resulting in a *B* value for the protein in a given formulation. Fig. 3 ▶ shows a comparison of the resulting *B* data for SIC and three different SLS studies (García, Holman *et al.*, 2003 ▶; Guo *et al.*, 1999 ▶; Rosenbaum & Zukoski, 1996 ▶). It is clear from these data that SIC is able to accurately measure *B* for lysozyme as a function of NaCl when compared with SLS. These data also suggest several important conclusions. The excellent correlation between the *B* values obtained by SIC and SLS indicate that that the immobilization chemistry used in SIC provides a set of random orientations mimicking the behavior of free proteins in solution. Secondly, SIC produces quantitatively similar data in much less time than SLS. The experiments shown here took approximately 20 min to generate a value for *B*, compared with several hours for each SLS data point. The possibility of protein precipitating in different solution conditions is generally not an issue, even for proteins that initially display very low solubility, since all conditions are run at relatively dilute protein concentrations (typically 1.0 mg ml^−1^). If for some particular protein immediate precipitation occurs upon mixing with the crystallization agent, one can simply reduce the protein concentration further until this situation is avoided.

**Figure 4:**
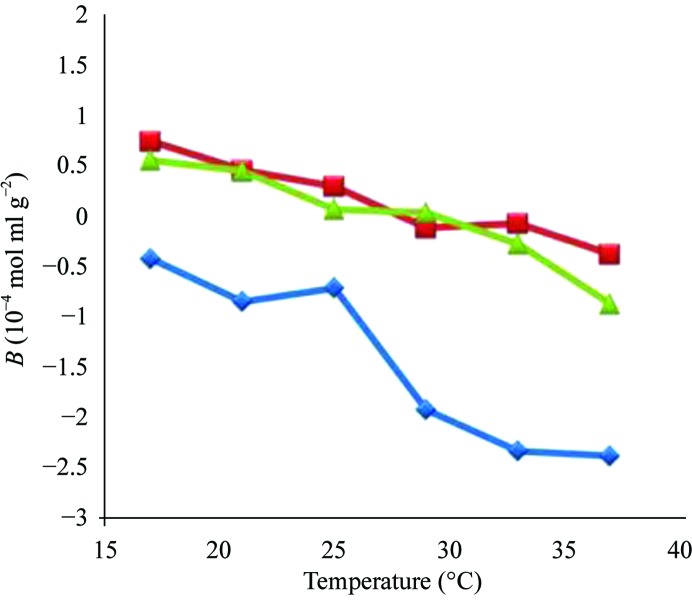
*B* as a function of ammonium sulfate concentration and temperature. Blue diamonds, no ammonium sulfate; red squares, 0.10 *M* ammonium sulfate; green triangles, 0.25 *M* ammonium sulfate.

The initial studies of the Wilson laboratory focused on lysozyme as a model protein, for which a wealth of *B* and solubility data is available. Lysozyme, however, is not an excellent model protein for studying self-interaction because it is very basic and very stable in its pure monomeric form. Fig. 4 ▶ shows *B* as a function of temperature and ammonium sulfate concentration for concanavalin A. Concanavalin A is well known to form dimers at low pH (below 7) and tetramers at high pH (above 7). The data shown in Fig. 4 ▶ are for the dimer system (verified by size-exclusion chromatography) and demonstrate the retrograde solubility of concanavalin A. This shows the ability to extend SIC to the measurement of complex multimeric proteins as well as accurately profiling the retrograde solubility of concanavalin A.

Comparisons of additional proteins using SIC were performed by Wilson as well as several other investigators. These studies showed the *B* values measured with SIC to closely compare with those using SLS, thereby establishing the use of SIC as an alternative method to measure second virial coefficients of proteins under different solution conditions (Tessier, Lenhoff *et al.*, 2002 ▶; García, Hadley *et al.*, 2003 ▶; Dumetz *et al.*, 2007 ▶; Patro & Przybycien, 1996 ▶; Pjura *et al.*, 2000 ▶; Valente *et al.*, 2005 ▶, 2006 ▶; DeLucas, 2009 ▶).

Crystallization is a stochastic event; thus, it is important that the protein to be crystallized is sufficiently concentrated to maximize the number of interactions between randomly oriented protein molecules (the vast majority of reported protein crystallization conditions range from 5.0 mg ml^−1^ to more than 100 mg ml^−1^; Tung & Gallagher, 2009 ▶). Protein aggregation is controlled by both conformational stability and colloidal stability, with either becoming the rate-limiting effect depending on solution conditions (Tsumoto *et al.*, 2005 ▶). Low solubility often produces nonspecific aggregation upon concentration, resulting in a nonhomogeneous population of protein aggregates (typically detrimental to the formation of high-quality crystals; Bergfors, 2009 ▶). Just as measured *B* values can be used to identify solution conditions that promote gentle protein–protein interactions, they can also be used to identify solution conditions that promote a net repulsion between protein molecules (positive *B* values). This capability addresses a significant problem for protein crystallo­graphers since many purified aqueous and membrane proteins exhibit low solubility.

Valente and coworkers investigated the use of SIC to measure *B* values for lysozyme in the presence of different cosolvents such as sucrose, trehalose, mannitol, glycine and arginine as well as combinations of these additives. Although each of the additives alone or in combination increased the *B* value (suggesting a reduction in intermolecular attraction), the magnitude of cosolvent-induced changes in the measured *B* value was found to be influenced by the control of long-range electrostatic repulsion. The work demonstrated that SIC provides an efficient, higher-throughput approach for obtaining measured *B* values of proteins in complex solutions. SIC has also been demonstrated to be a sensitive technique. For example, SIC is able to discriminate site-directed mutations of the surface amino acids of a protein provided that they do, in fact, affect protein–protein interactions (Wilson *et al.*, 2009 ▶). These initial studies suggest that SIC can be used to provide quantitative data to guide protein surface molecular engineering to support protein solubility and physical stability (important characteristics for several biological research disciplines). It can also be used to identify changes in surface residues that result in *B* values that lie within the crystallization slot. Studies also demonstrated how SIC can be used to explore and optimize different combinations and concentrations of mixed detergent systems for specific membrane proteins (Wilson *et al.*, 2009 ▶). Using proteorhodpsin (pR), a member of the rhodopsin protein family, SIC was performed to explore the effect of pH, surfactant concentration and surfactant type on the biological activity of pR. For this particular membrane protein, when two non-ionic detergents were mixed the conformational stability was dependent on the detergent type and concentration. SIC was used to produce contour plots with the concentrations of each detergent plotted along the *x* and *y* axes and the biological activity of pR (monitored *via* the absorbance at 531 nm) representing the third dimension.

**Figure 5:**
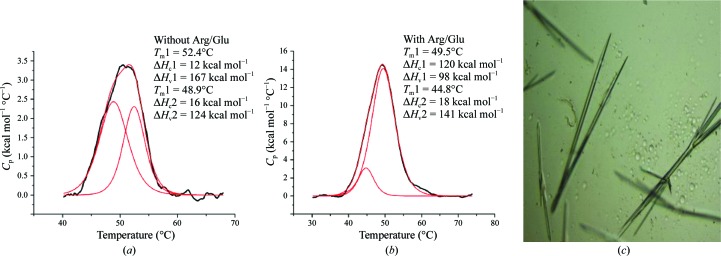
(*a*, *b*) DSC scans of NaMNAT protein dissolved in different additive solutions. (*a*) NaMNAT at 0.324 mg ml^−1^ in buffer *A* (50 m*M* Tris–HCl pH 7.5, 100 m*M* NaCl, 10% glycerol, 1 m*M* DTT). *B* value = −9.6. (*b*) NaMNAT at 8.7 mg ml^−1^ in buffer *B* (50 m*M* Tris–HCl pH 7.5, 100 m*M* NaCl, 10% glycerol, 1 m*M* DTT, 50 m*M* arginine, 40 m*M* glutamic acid, 20 m*M* trehalose). *B* value = +1.6 × 10^−4^ mol ml g^−2^. (*c*) Crystals of NaMNAT prepared with PEG 8000 and malonate as precipitant agents from the solution in (*b*) containing 50 m*M* arginine, 40 m*M* glutamic acid and 20 m*M* trehalose. Polydispersity = 0.035. Crystallization *B* value = −5.1 × 10^−4^ mol ml g^−2^.

One example of the use of SIC to improve the solubility and crystallization of a protein was reported by Lu *et al.* (2008 ▶). In this case, improvements in homogeneity and solubility were necessary for the crystallization of a totally new, not previously crystallized protein, *Bacillus anthracis* nicotinate mononucleotide adenylyltransferase (*Ba*-NaMNAT). Nicotinic acid mononucleotide adenylyltransferase (NaMNAT) is the penultimate enzyme in the biosynthesis of NAD^+^ and catalyzes the adenylation of nicotinic acid mononucleotide by ATP to form nicotinic acid adenine dinucleotide (NaAD). *Ba*-NaMNAT, a suitable candidate for antibacterial drug development, was heterologously expressed in *E. coli* for the purpose of inhibitor discovery and crystallography. The expressed protein was purified using metal-chelation chromatography, yielding protein at 2.7 mg ml^−1^, which was subsequently dialyzed against buffer *A* (composition given in Fig. 5 ▶ *a*), after which the majority of the protein precipitated. Only approximately 0.3–0.4 mg ml^−1^ protein remained in solution. Fig. 5 ▶(*a*) shows a DSC scan of the remaining soluble protein. The ratio of the van’t Hoff to calorimetric enthalpy is an indication of the cooperativity of the transition. After deconvolution, the ratio for the two peaks is much in excess of a ratio of 6 to 14 (data not shown), indicative of either an aggregated or a partially unfolded protein. The second virial coefficient value for the protein dissolved in buffer *A* was −9.6 × 10^−4^ mol ml g^−2^, indicating significant protein–protein attraction. A second purification was conducted in the same buffers but with the addition of 50 m*M* arginine and 50 m*M* glutamic acid. This time the protein was easily soluble up to 5 mg ml^−1^ when dialyzed into buffer *A* (the *B* value for buffer *A* with 50 m*M* arginine and 50 m*M* glutamic acid is +1.6 × 10^−4^ mol ml g^−2^, indicating slight protein–protein repulsion). The DSC shown in Fig. 5 ▶(*b*) revealed a main transition with a ratio for the van’t Hoff to calorimetric enthalpy of close to unity (0.82), suggestive of a monomeric protein, with only a small amount of aggregate present (a peak of less than 17% at 44.8°C based on enthalpy). The differences in *T*_m_ between Figs. 5 ▶(*a*) and 5 ▶(*b*) are owing to the presence of arginine and glutamic acid additives.

**Figure 6:**
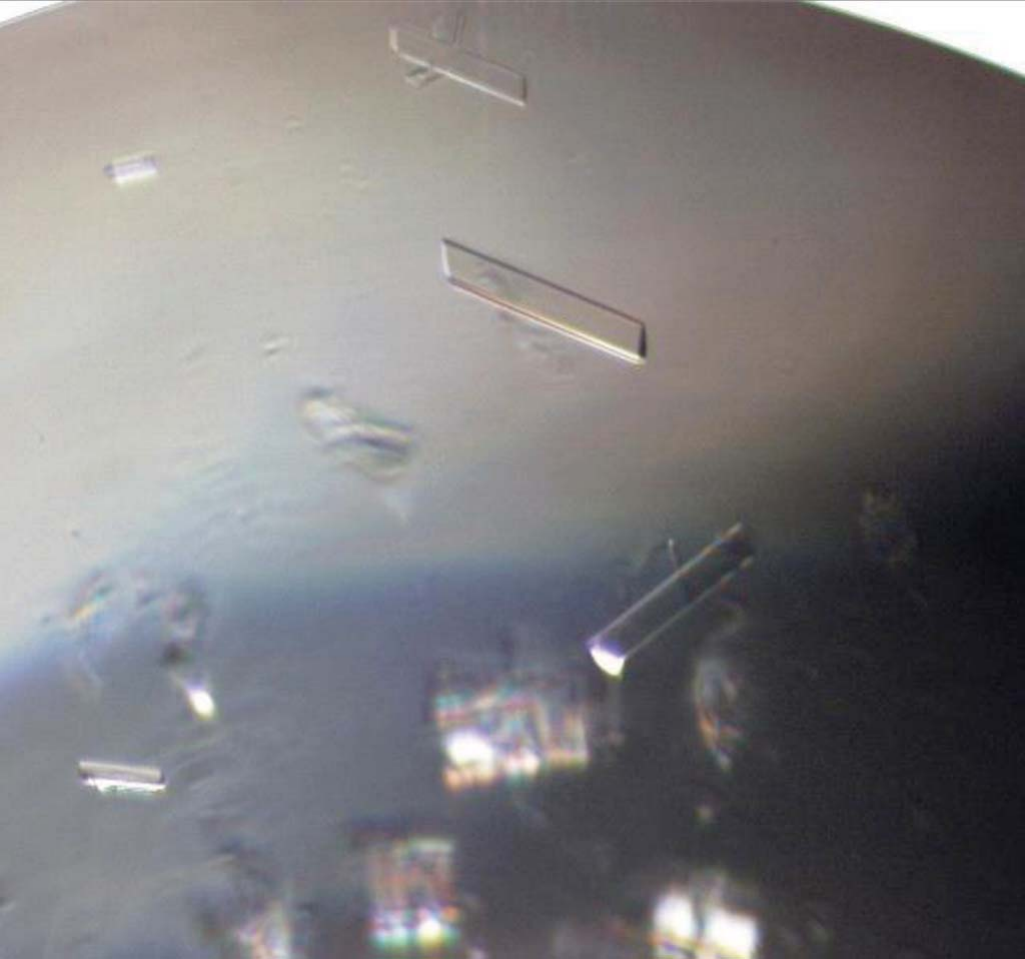
NaMNAT crystals (0.40 × 0.07 × 0.02 mm).

Further refinement of additives and their respective concentrations using SIC yielded the following final conditions: buffer *A* with 100 m*M* Glu, 100 m*M* Arg and 200 m*M* trehalose; final *Ba*-NaMNAT concentration of 12.1 mg ml^−1^, *B* value = +2.7 × 10^−4^ mol ml g^−2^, polydispersity = 0.028. Crystallization screening was initiated with this monodisperse sample [judged by a polydispersity of 0.028 *via* dynamic light-scattering (DLS) experiments] with a solubility exceeding 12.0 mg ml^−1^. This solution condition was used as the common protein buffer for subsequent high-throughput crystallization screens. Refinement of the conditions involved varying the percentages of precipitant in subsequent crystallization experiments followed by SIC to determine how the additives were changing the *B* value with respect to the crystallization slot. The final solution crystallization conditions were also within the crystallization slot but resulted in a slightly less negative *B* value of −4.7 *versus* −5.1 × 10^−4^ mol ml g^−2^ for the conditions yielding the needle-like crystal morphology. The final crystallization conditions yielded high-quality crystals (Fig. 6 ▶) from which a 2.3 Å resolution X-ray structure was determined. This example demonstrates the value of this knowledge-based approach. A similar iterative approach was used for the membrane protein light-harvesting complex 1 reaction center core complex from *Allochromatium vinosum* (Gabrielsen *et al.*, 2010 ▶).

Compelling examples of the use of *B* values for membrane-protein crystallization have been reported in several publications (Kratochvil, 1987 ▶; Tsumoto *et al.*, 2005 ▶; Berger *et al.*, 2005 ▶, 2006 ▶; Bhat & Timasheff, 1992 ▶). As noted earlier, before the advent of self-interaction chromatography for *B* determinations it was extremely difficult to obtain such measurements *via* the traditional approach of static light scattering. The solution behavior of the bacterial outer membrane protein OmpF porin was studied by SLS in a variety of crystallization solutions (Tsumoto *et al.*, 2005 ▶). *B* was demonstrated to be a clear predictor of the crystallization behavior of porin. Both tetragonal and trigonal porin crystals were found to form in a narrow range of *B* values which were only located within the ‘crystallization slot’ as defined by Wilson for aqueous proteins. *B* values were also used to study the effect of precipitants such as PEG on micelle size and interaction forces between micelles (Kratochvil, 1987 ▶; Tsumoto *et al.*, 2005 ▶).

*B* values for detergent micelles (free of protein) under identical crystallization conditions exhibited similar *B* values to the protein–detergent complexes (PDCs). Thus, based on this one example using OmpF, in which the detergent appears to dominate the interactions between PDCs, the authors suggest that for any given detergent membrane-protein crystallization screens may be designed by simply manipulating the detergent-solution properties until the measured *B* values lie within the crystallization slot. This would minimize the amount of protein required for crystallization screening and improve productivity.

Studies performed by Berger and coworkers used measured second virial coefficient values to investigate protein interactions, leading to the crystallization of bacteriorhodopsin solubilized in *n*-octyl-β-d-glucoside (Berger *et al.*, 2006 ▶). At low to moderate salt concentrations the PDC *B* values show an increase in repulsive interactions followed by a sharp transition to attractive PDC interactions in a narrow range of high salt concentrations. PDC inter­actions ‘were observed as the cloud-point temperature was approached for various salts’, suggesting that the interaction trends are strongly influenced by the micelle structure and surfactant phase behavior, both of which are sensitive to salt and surfactant concentration. These data also suggest that for a PDC solution at a fixed surfactant concentration above its CMC the surfactant interactions may play a significant role in PDC interactions. Micelle shape and structure (influenced by the micelle concentration in specific salt concentrations) are also demonstrated to be important in PDC interactions. It was also demonstrated that for various combinations of additives and precipitants studied, attractive PDC interactions tend to occur at conditions approaching the C_8_βG_1_ cloud curve (the cloud point is the temperature or other variable change at which dissolved solids begin to precipitate, producing a cloudy appearance to the solution). This was observed previously for OmpF (Tsumoto *et al.*, 2005 ▶).

Another example of the use of the second virial coefficient for membrane-protein crystallization involved 30 *B*-value measurements by Berger and coworkers for the bacteriorhodopsin (bR) PDC over a wide range of solution conditions at which a corresponding cloud-point temperature was measured. A strong correlation was observed between the measured *B* value of the bR C_8_βG_1_ PDCs and the corresponding cloud-point temperature of C_8_βG_1_. Crystallization trials resulted in a subset of these conditions where crystallization occurred in the ‘crystallization slot’ as defined by Wilson for aqueous proteins. It was also apparent that surfactant phase behavior and interactions play a significant role in promoting PDC crystallization. Within the range of salt concentrations used in bacteriorhodopsin crystallization, the CMC of β-octyl-d-glucoside (C_8_βG_1_) decreases by at least an order of magnitude. Lorber, DeLucas and Bishop observed similar results previously (Arakawa & Timasheff, 1985 ▶). Berger’s paper clearly demonstrated that the second virial coefficient combined with other techniques can provide insight into the complex nature of PDC interactions, information which is important to developing rational approaches to membrane-protein crystallization. Berger’s example with bacteriorhodopsin suggests that the second virial coefficient can initially be used to map out the crystallization slot for a specific protein followed by the adjustment of initial pre-crystallization conditions (detergent, salt and other additive concentrations) to produce more PDC–PDC interactions, leading to a wider range of weakly attractive *B* values. Alterations in the initial solution conditions based on measured *B* values can potentially provide diagnostic information to enable a more gentle transition into the crystallization slot (Berger *et al.*, 2005 ▶, 2006 ▶).

The use of self-interaction chromatography (SIC) to measure second virial coefficients provides several benefits compared with other *B*-value methods, including the following: (i) precision HPLC, a widely used instrumentation, is all that is needed to perform SIC; (ii) SIC measurements are relatively easy to perform, requiring minimal time and user expertise (each measurement requires less than 30 min); (iii) *B* values are directly correlated to protein solubility and solution conditions that support crystallization; (iv) a choice of simple chemistries can be used to covalently couple protein in random orientations to column media; (v) SIC can be performed on both aqueous and membrane proteins; and (vi) SIC can be easily automated. However, SIC, as introduced in 1996 by Patro and Przybycien, required 28 mg of purified protein to prepare a 1.6 ml column of Sepharose gel. Improvements in SIC chromatographic accuracy and protein coupling chemistry have been reported by Tessier and coworkers (Tessier, Lenhoff *et al.*, 2002 ▶; Tessier, Vandrey *et al.*, 2002 ▶), reducing the quantity of protein required to 6.5 mg for a 1 ml column of Toyopearl AF-Formyl particles (Tosoh BioScience). More recently, Wilson and coworkers developed a microchip for SIC using poly(dimethylsiloxane) (PDMS), providing a 500-fold reduction in protein consumption (García, Hadley *et al.*, 2003 ▶).

**Figure 7:**
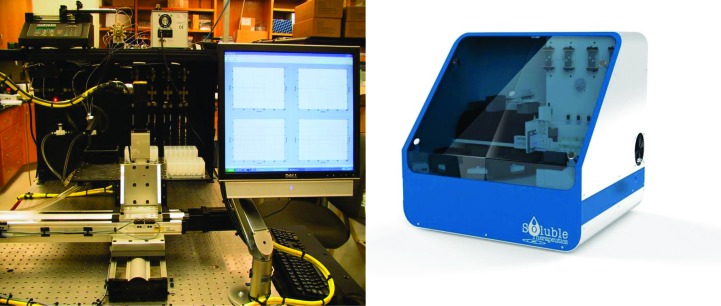
Second-generation HSC (left) and third-generation HSC (right).

**Figure 8:**
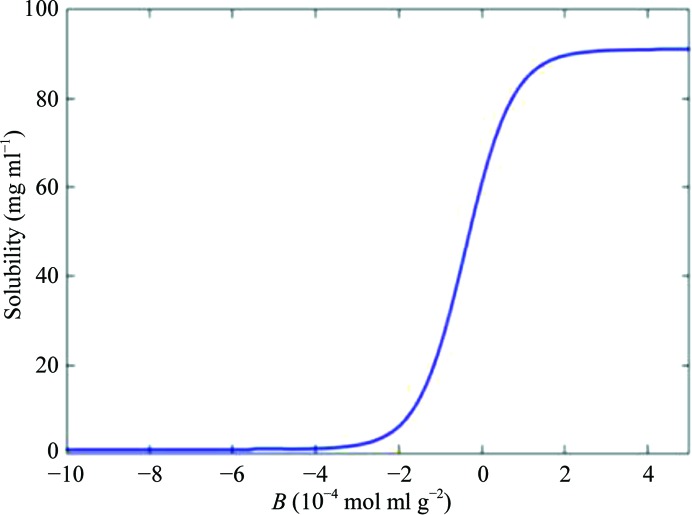
Protein solubility *versus* *B*.

**Figure 9:**
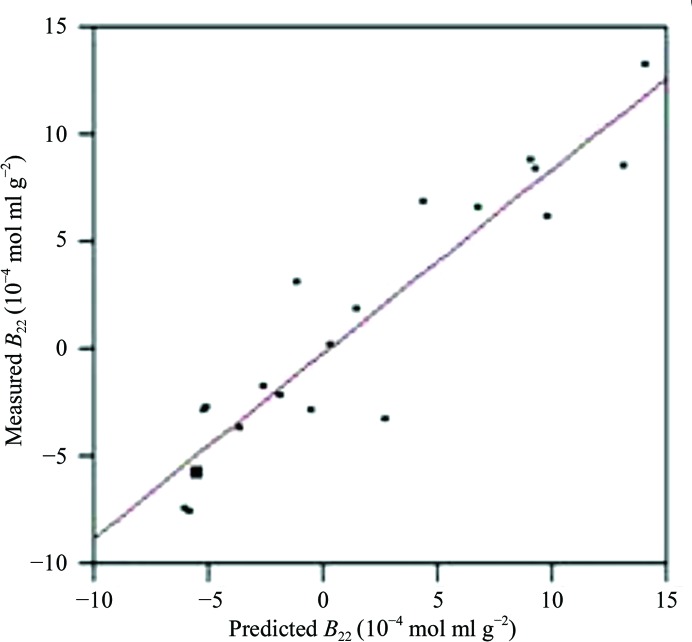
Predicted *versus* observed *B* values for excipient screen (*N* = 20, RMSE = 2.50).

**Figure 10:**
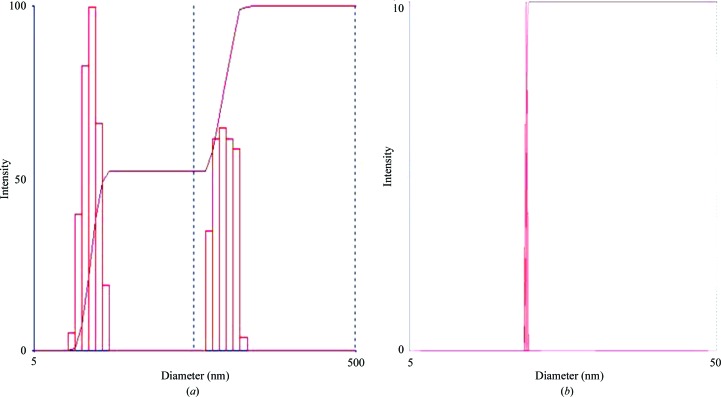
The aggregation of hOPG decreases with the addition of additives. Particle size distribution determined by DLS analysis of hOPG purified (*a*) in the absence of additives (% cumulative polydispersity index = 0.32) and (*b*) in the presence of 0.1 *M* l-arginine and l-glutamic acid, 0.2 *M* trehalose and 2 m*M* DTT (% cumulative polydispersity index = 0.10).

**Figure 11:**
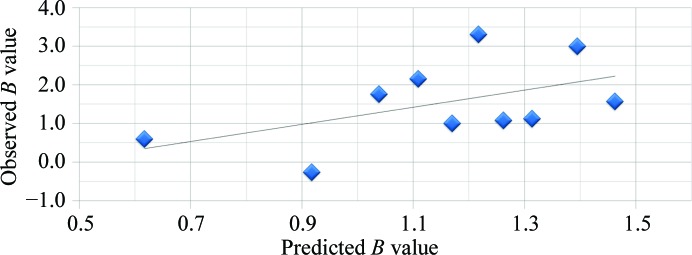
Observed *versus* predicted *B* values (10^−4^ mol ml g^−2^) for osteoprotegerin.

Fig. 7 ▶ shows two generations of robotic SIC systems (HSC), with the first- and second-generation systems being developed from 2006 to 2010 at the University of Alabama at Birmingham and the third-generation system developed by a biotechnology company (Soluble Therapeutics Inc.). The HSC system includes the following improvements: (i) a significant reduction in the amount of protein required to 0.8 mg *via* the use of a column with an internal diameter of 0.54 mm and a length of 180 mm; (ii) increased throughput *via* the simultaneous operation of four independent columns; and (iii) automated formulation and protein injection. A detailed description of the HSC has recently been submitted for publication. However, even this fully automated higher throughput system is not sufficiently fast to screen hundreds to thousands of solution conditions (protein consumption would still be problematic if a thousand or more conditions were screened). Additionally, it is difficult and sometimes impossible for a trained crystallographer to identify subtle or hidden crystallization trends for a large set of experiments each containing different solution components/conditions. For this reason, Wilson and DeLucas investigated the use of measured *B* values for a subset of conditions or an incomplete factorial (representing the range of conditions and component combinations of the complete crystallization screen). The incomplete factorial conditions and their corresponding measured *B* values are input to an artificial neural network (ANN) that ‘trains itself’ and subsequently predicts a full factorial of solution conditions and corresponding *B* values (Wilson *et al.*, 2009 ▶). The combined use of an automated SIC system and ANN (referred to as the HSC system) provides multiple advantages including (i) higher throughput for experimentally determined *B* values; (ii) reasonably low protein consumption owing to the small column size; and (iii) reduction of the number of initial conditions that must be surveyed to provide sufficient information for the ANN to accurately predict the full factorial of conditions. The ANN provides a more efficient method (in terms of time and protein consumption) to identify novel solution combinations that provide slight protein–protein attraction (*i.e.* those conditions with *B* values within the crystallization slot). The approach of using an incomplete factorial (Kendrick *et al.*, 1997 ▶) of crystallization conditions and outcomes combined with ANN analysis has previously been reported by DeLucas and coworkers (DeLucas *et al.*, 2003 ▶, 2005 ▶). The ability to improve or maximize the solubility of purified proteins is beneficial to many different protein research disciplines. One major application involves protein therapeutics, a discipline often requiring concentrations exceeding 100 mg ml^−1^. The formulation divisions of pharmaceutical companies evaluate hundreds of combinations of small-molecule additives (termed excipients) in an effort to find specific additive combinations and concentrations that improve the solubility and physical stability (aggregation) of protein therapeutics. There are two general classes of excipients: those that block hydrophobic patches (*i.e.* arginine and glutamic acid) on the surface of proteins, thereby decreasing nonspecific aggregation, and those that stabilize protein conformation (*i.e.* trehalose, sorbitol and mannitol) *via* their influence on the organization of and properties of solvent water. Other variables that can affect solubility of aqueous or membrane proteins include salt type/concentration, pH, temperature, reducing agents, type/concentration of detergent and other surfactants (Tsumoto *et al.*, 2005 ▶; Arakawa & Timasheff, 1985 ▶; Bhat & Timasheff, 1992 ▶; Kendrick *et al.*, 1997 ▶; Chi *et al.*, 2003 ▶; Kaushik & Bhat, 2003 ▶; Lins *et al.*, 2004 ▶; Timasheff, 1998 ▶; Valente *et al.*, 2005 ▶). As noted previously, measured *B* values provide a qualitative assessment of protein–protein interactions, with more positive or more negative *B* values indicative of increased repulsion or increased attraction, respectively. The *B*-value measurement by HSC provides protein–protein interaction information in every formulation condition tested. However, there remains the problem of the dimensionality of an exhaustive search of parameters. ANNs have been successfully used for pattern recognition in high-dimensional parameter space (Chi *et al.*, 2003 ▶). The ANN, as is the case for traditional linear regression models, is a method of mapping input parameters (formulation variables) to an output variable (*B* value). Measured *B* values for aqueous and membrane proteins provides valuable information to improve/optimize aqueous and membrane protein solubility, physical stability and crystallization. However, there are a large number of different variables and variable concentrations that must be explored. It is important that a representative set of conditions are investigated so as not to miss important combinations/concentrations that might improve the solubility, homogeneity or stability of a protein or its ability to produce high-quality crystals. At least 25 factors can influence the formation of protein crystals (Kaushik & Bhat, 2003 ▶). In addition, each factor can include different values, thereby multiplying the total number of factors that must be evaluated. The category of additives can include hundreds of different compounds, each with its own concentration variable. Thus, an exhaustive complete factorial search of all possible variable combinations is not possible. By combining ANN analysis of an incomplete factorial screen that surveys multiple parameter combinations and concentrations, the total number of screening conditions can be dramatically reduced. The use of an ANN and incomplete factorial screens have proven to be effective in crystallization, although the predicted novel crystallization conditions typically resulted in a large number of false positives (DeLucas *et al.*, 2003 ▶, 2005 ▶). However, the application of ANN and incomplete factorial solubility screens has proven to be more promising. The correlation between *B* and protein solubility has been presented both experimentally and theoretically for several proteins (Demoruelle *et al.*, 2002 ▶; Gripon *et al.*, 1997 ▶; Haas *et al.*, 1999 ▶; Malfois *et al.*, 1996 ▶; Neal *et al.*, 1998 ▶; Ruppert *et al.*, 2001 ▶). Fig. 8 ▶ shows the general relationship between *B* and *s* (a typical solubility curve). It was previously demonstrated that *B* values can be predicted with a high level of accuracy by an ANN model trained on a small set of *B*-value measurements. The first demonstration included only 81 measured formulation conditions that were used to train an ANN model. The model was then used to predict the *B* values for 12 626 formulations. 20 of the formulations were verified experimentally, and the predictions were found to have an error rate somewhat larger than the error inherent to SIC *B*-value measurements, but the results were clearly semi-quantitative in predicting *B* values for unmeasured formulations (Fig. 9 ▶). In the first stage, individual additives are screened to identify which additives are most effective at reducing protein–protein interactions as measured by the second virial coefficient *B*. There are as many as 300 additive formulations to choose from that are approved by the FDA for use in drug formulations (http://www.accessdata.fda.gov/scripts/cder/iig/index.cfm). The choice of any two (of the 300) additives at one of three concentration levels would result in over 404 550 possible formulation conditions. The initial screen identifies which additives individually reduce protein–protein interactions (*i.e.* more positive *B* values). Those additives demonstrated to not adversely affect protein stability (*via* DSC analysis) are combined into formulations based on a design of experiments using an orthogonal array (assuring that combinations of additives are equally represented throughout the screen). For each protein studied, a numerical model of how additives affect protein–protein interaction (*B* value) is created by training an ANN using experimental data generated from the screen. The ANN is then used to predict *B* values for the full factorial of screened additives. Development of human osteoprotegerin (hOPG) mimetics has been suggested as a possible strategy for the treatment of diseases such as osteoporosis (López, 2000 ▶). hOPG is difficult to purify sufficiently for crystallization experiments owing to its poor solubility and tendency to aggregate. SIC was used to determine *B* values for an excipient screen followed by ANN analysis. The results showed an excellent correlation between the predicted *versus* experimentally measured *B* values, with several predicted excipient combinations resulting in improved (more positive) *B* values compared with the control solution. In addition, several of the predicted excipient combinations resulted in substantial solubility improvements. For example, 100 m*M* l-arginine, 100 m*M* l-glutamic acid, 200 m*M* trehalose and 2 m*M* DTT (*B*_meas_ = +2.1 × 10^−4^ mol ml g^−2^) improved the solubility beyond 10 mg ml^−1^. DLS analysis of the protein in this excipient mixture suggested that it significantly reduced the aggregation state of hOPG in solution (Fig. 10 ▶). Comparison of the observed *versus* predicted *B* values (Fig. 11 ▶) for ten of the top predictions shows a reasonable correlation. Note that for this protein the changes in *B* values are not large, but are consistent with Fig. 8 ▶, which shows that small changes in *B* can correspond to significant changes in solubility, especially at higher concentrations. All of these formulations resulted in a shift in *T*_m_ to higher temperatures compared with hOPG in base buffer (the highest shift was 74.7°C compared with 63.9°C). The formulations for the two highest measured *B* values were (i) 100 m*M* MES pH 6.1, 300 m*M* NaCl, 150 m*M* Arg, 150 m*M* Glu, 3%(*v*/*v*) hexanediol and (ii) 100 m*M* Tris pH 8.2, 5 m*M* MgSO_4_, 150 m*M* Arg, 100 m*M* Glu, 6%(*v*/*v*) hexanediol.

**Figure 12:**
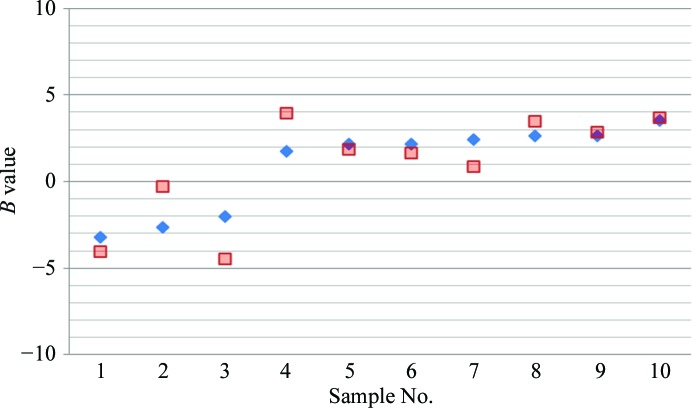
CFTR NBD1 *B*-value measurements (10^−4^ mol ml g^−2^) (red squares) *versus* neural net predictions (blue diamonds).

**Figure 13:**
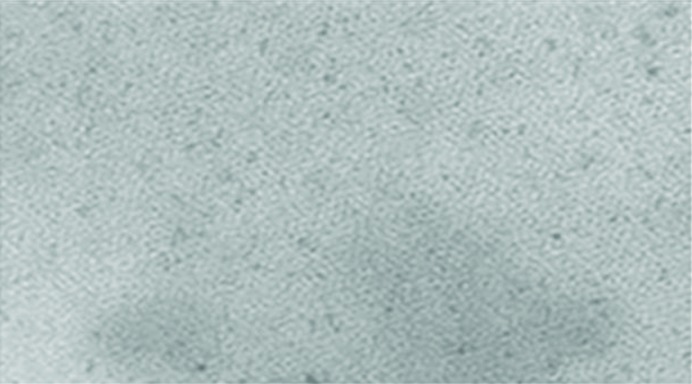
Electron micrograph of purified full-length chicken CFTR dissolved in 1.0 m*M* ATP, 50 m*M* sodium phosphate, 1.0 *M* lithium chloride, 1 m*M* DDM.

In spite of selection from a variety of protein-expression systems, the expression and purification of protein in milligram quantities remains an impediment for many proteins, especially integral membrane proteins (IMPs). Although progress has been made in scaling down the protein consumption of SIC, the typical protein still requires ∼15 mg of purified protein to prepare several columns to support an incomplete factorial analysis followed by validation of a subset of ANN predictions (the preparation of each SIC column generally requires 1.0 mg purified protein). However, there is an alternative approach that may be useful for integral membrane proteins or any protein that is difficult to express. In many cases, specific domains of a protein may be isolated and expressed in larger quantities to support an HSC analysis (*i.e.* extracellular or intra­cellular domains associated with IMPs). An example of this approach has been reported for the cystic fibrosis transmembrane regulator protein (CFTR), an integral membrane protein that is extremely difficult to express in milligram quantities, displays low solubility and has a tendency to form larger aggregates. CFTR is composed of five domains, two of which are nucleotide-binding domains that exhibit poor solubility when individually expressed and purified. Milligram quantities of nucleotide-binding domain 1 (NBD1) were expressed and purified for subsequent HSC analysis. The NBD1 domain is one of three domains suspected to be responsible for the poor solubility of the full-length membrane protein. The predicted HSC conditions with the highest positive *B* values were experimentally validated and subsequently used to assess the solubility of full-length CFTR. These studies were performed in collaboration with Dr Robert Ford at Manchester University. The excellent correlation between the ANN predicted *B* values *versus* experimental measurements in these same solution conditions are shown in Fig. 12 ▶ for NBD-1. The highest positive *B* values were observed for high ionic strength solutions (particularly for lithium chloride as opposed to other monovalent or divalent salts). Solution conditions exhibiting the most positive *B* values for NBD1 were then used to purify full-length chicken CFTR, resulting in a tenfold improvement in solubility (from 0.05 to 0.5 mg ml^−1^). The electron micrograph (Fig. 13 ▶) shows a homogeneous preparation of purified full-length chicken CFTR in solution conditions resulting from HSC technology.

## 3. Summary   

HSC technology provides a number of advantages over other methods used to determine second virial coefficients including the following: (i) it can be performed using a precision HPLC chromatography system as present in many academic and industrial bio-laboratories, with each experiment requiring less than 30 min; (ii) there are four alternative protein coupling chemistries, thereby providing alternatives if one or more has an adverse effect for a particularly sensitive or labile protein; (iii) compared with other methods used to determine second virial coefficients, self-interaction chromatography requires relatively little experimenter expertise to prepare the column and protein, to operate an HPLC and to calculate *B* values from the experimental data generated; (iv) it can be used to improve solubility for aqueous and membrane proteins dissolved in solvents containing different additives and/or different detergents/lipids; and (v) as described in this review, self-interaction chromatography is amenable to automation and higher throughput. Possible limitations to use of this technique include the following. (i) The protein concentration covalently coupled to the column medium must be high enough to ensure a sufficient number of protein–protein interactions to provide accurate measured *B* values. In most cases, the protein must exhibit a minimum solubility of 3.0 mg ml^−1^ prior to chemical coupling. For proteins exhibiting lower initial solubilities, the column can be lengthened in an effort to increase the number of protein–protein interactions. (ii) Larger proteins exhibit fewer interactions with protein bound to the column medium than smaller proteins bound at the same concentration, decreasing the accuracy of the measured *B* values (this is also countered by increasing the column length). (iii) High-viscosity solvents may increase the column back pressure to the point where the column medium is compressed, thereby broadening the chromatographic peaks and decreasing the accuracy of the measured *B* values. However, in spite of these possible limitations, the previous studies presented in this article demonstrate the usefulness of self-interaction chromatography as an alternative method for measuring protein–protein interactions.

The HSC technology combined with incomplete factorial solubility screens and ANN analysis has led to solubility improvements ranging from less than 1.0 mg ml^−1^ to as high as 160 mg ml^−1^ (submitted). Efforts are currently under way to further reduce the dimensions of SIC columns in an effort to facilitate use of this technology for difficult-to-express proteins. The studies presented in this article demonstrate the usefulness of self-interaction chromatography for a variety of applications. It is anticipated that the combination of the higher throughput automated SIC system with the ability to screen a reasonably small number of solution conditions will ultimately prove beneficial to protein research performed in both academia and industry.
